# A new stress sensor and risk factor for suicide: the T allele of the functional genetic variant in the GABRA6 gene

**DOI:** 10.1038/s41598-017-12776-8

**Published:** 2017-10-10

**Authors:** Xenia Gonda, Jane Sarginson, Nora Eszlari, Peter Petschner, Zoltan G. Toth, Daniel Baksa, Gabor Hullam, Ian M. Anderson, J. F. William Deakin, Gabriella Juhasz, Gyorgy Bagdy

**Affiliations:** 1 MTA-SE Neuropsychopharmacology and Neurochemistry Research Group, Hungarian Academy of Sciences, Semmelweis University, Budapest, Hungary; 2 Department of Psychiatry and Psychotherapy, Kutvolgyi Clinical Centre, Semmelweis University, Budapest, Hungary; 3 NAP-A-SE New Antidepressant Target Research Group, Semmelweis University, Budapest, Hungary; 4 School of Health Sciences, University of Manchester, Manchester, United Kingdom; 5 School of Healthcare Science, Manchester Metropolitan University, John Dalton Building, Chester Street, Manchester, M15GD UK; 6 Department of Pharmacodynamics, Faculty of Pharmacy, Semmelweis University, Budapest, Hungary; 7 Institute of Communication Engineering, Kando Kalman Faculty of Electrical Engineering, Obuda University, Budapest, Hungary; 8 MTA-SE-NAP B Genetic Brain Imaging Migraine Research Group, Hungarian Academy of Sciences, Semmelweis University, Budapest, Hungary; 9 Department of Measurement and Information Systems, Budapest University of Technology and Economics, Budapest, Hungary; 10 Neuroscience and Psychiatry Unit, Division of Neuroscience and Experimental Psychology, University of Manchester and Manchester Academic Health Sciences Centre, Manchester, United Kingdom; 11 Greater Manchester Mental Health NHS Foundation Trust, Manchester, UK

## Abstract

Low GABA transmission has been reported in suicide, and *GABRA6* rs3219151 T allele has been associated with greater physiological and endocrine stress response in previous studies. Although environmental stress also plays a role in suicide, the possible role of this allele has not been investigated in this respect. In our present study effect of rs3219151 of *GABRA6* gene in interaction with recent negative life events on lifetime and current depression, current anxiety, as well as lifetime suicide were investigated using regression models in a white European general sample of 2283 subjects. Post hoc measures for phenotypes related to suicide risk were also tested for association with rs3219151 in interaction with environmental stress. No main effect of the *GABRA6* rs3219151 was detected, but in those exposed to recent negative life events *GABRA6* T allele increased current anxiety and depression as well as specific elements of suicide risk including suicidal and death-related thoughts, hopelessness, restlessness and agitation, insomnia and impulsiveness as measured by the STOP task. Our data indicate that stress-associated suicide risk is elevated in carriers of the *GABRA6* rs3219151 T allele with several independent markers and predictors of suicidal behaviours converging to this increased risk.

## Introduction

Every year suicide accounts for one million deaths worldwide (nearly 2% of all deaths), amounting to one every 40 seconds, a mortality rate of 16/100,000^1^. Suicide and suicidal behaviour are not diagnosis-specific but a symptom and are a feature of many psychiatric disorders^2^. The diathesis for suicide includes a genetic predisposition with heritability of suicide estimated at about 55%^3^ and a similar degree of heritability for nonfatal suicidal acts^4^. Familial and adoption studies indicate that transmission of suicidal behaviour is independent of Axis I and II disorders^5,6^ and is also influenced by developmental and rearing conditions. An accumulation of stressful and traumatic life events, losses, and acute and chronic somatic and mental illnesses contribute to neurobiological alterations^3,7^ emphasising the role of stress in the emergence of suicide.

As the major inhibitory neurotransmitter in humans, GABA plays an important role in downregulating HPA-axis in response to acute stress as demonstrated by the strong inhibitory effect of alprazolam on HPA-axis activation following experimental stress^8^. Exposure to stress can have short- and long-term effects in the GABA system including altering the availability of GABA-A receptors as well as their composition and sensitivity to neurosteroid regulation which can in turn result in an altered response to subsequent or ongoing stressors^9–12^. Rs3219151 in *GABRA6* appears to have a modulatory effect on HPA-axis activity as demonstrated by an association between the T allele and higher plasma cortisol levels both at rest^13^ and also when stimulated during the Trier Social Stress Test^14^ suggesting this allele may increase the stress response.

Expression of several GABA-A receptor subunits has been shown to be strikingly upregulated across several brain regions in a large series of post mortem brains of suicide victims compared to controls^15^. The global change raises the possibility that genotypic variation may underpin these changes. In spite of its association with stress reactivity, the *GABRA6* T allele has so far not been investigated in relation to suicidal behaviour. Gene expression studies in post mortem brain strongly implicating abnormal GABA function^16,17^ led us to explore phenotypes for suicide risk markers associated with this variant. In the present study we carried out exploratory analyses of association of *GABRA6* rs3219151 with symptomatic and pathogenic risk factors of suicide in interaction with recent stressors in a large European nonpsychiatric population.

## Results

No associations between genotype and demographic or lifestyle measures were identified. The Budapest and Manchester cohorts differ significantly for demographics (except for gender), exposure and outcome measures (Table 1). The Manchester cohort reported a higher level of recent negative life events (RLE). They also have higher levels of current depression (BSI-DEP) and current anxiety (BSI-ANX) symptoms, and higher rate of self-reported lifetime depression (DEP) and self-reported lifetime suicide attempt or deliberate self-harm (SUIC).

**Table 1:** Description of the Level 1 study populations.

		Total population	Cohort		Comparison (p-value)
			Budapest	Manchester	
**Population size**	(N)	2283	975	1308	
**Demographics**					
Gender	(% Male)	30.80%	31.40%	30.40%	0.597
Age	(Mean ± SEM) (range)	32.86 ± 0.223 (18–60)	31.22 ± 0.352 (18–60)	34.02 ± 0.286 (18–60)	<0.0001
**Adversity scores**					
Sum recent negative life events	(Mean ± SEM)	1.22 ± 0.027	1.08 ± 0.038	1.32 ± 0.038	<0.0001
**Lifetime depression**					
BGR lifetime depression	(%)	41.30%	21.40%	56.20%	<0.0001
**Recent depression/anxiety**					
Current BSI depression score	(Mean ± SEM)	0.8 ± 0.019	0.5 ± 0.022	1.07 ± 0.028	<0.0001
Current BSI anxiety score	(Mean ± SEM)	0.8 ± 0.018	0.6 ± 0.023	1.02 ± 0.027	<0.0001
**BSI-DEP items**					
Thoughts of ending your life	(Mean ± SEM)	0.36 ± 0.019	0.25 ± 0.025	0.45 ± 0.027	<0.0001
Poor appetite	(Mean ± SEM)	0.55 ± 0.021	0.42 ± 0.028	0.65 ± 0.029	<0.0001
Feeling lonely	(Mean ± SEM)	1.19 ± 0.029	0.88 ± 0.040	1.42 ± 0.039	<0.0001
Feeling blue	(Mean ± SEM)	1.26 ± 0.028	0.93 ± 0.037	1.50 ± 0.039	<0.0001
Feeling no interest in things	(Mean ± SEM)	0.82 ± 0.026	0.40 ± 0.029	1.13 ± 0.038	<0.0001
Trouble falling asleep	(Mean ± SEM)	1.05 ± 0.029	0.67 ± 0.036	1.33 ± 0.040	<0.0001
Feeling hopeless about the future	(Mean ± SEM)	0.97 ± 0.028	0.64 ± 0.035	1.21 ± 0.039	<0.0001
Thoughts of death or dying	(Mean ± SEM)	0.70 ± 0.025	0.52 ± 0.033	0.82 ± 0.035	<0.0001
Feelings of worthlessness	(Mean ± SEM)	0.79 ± 0.026	0.39 ± 0.027	1.09 ± 0.039	<0.0001
Feelings of guilt	(Mean ± SEM)	0.87 ± 0.026	0.54 ± 0.030	1.11 ± 0.037	<0.0001
**BSI-ANX items**					
Nervousness or shakiness inside	(Mean ± SEM)	1.29 ± 0.026	1.34 ± 0.040	1.26 ± 0.035	0.068
Suddenly scared for no reason	(Mean ± SEM)	0.64 ± 0.022	0.48 ± 0.030	0.76 ± 0.032	<0.0001
Feeling fearful	(Mean ± SEM)	0.84 ± 0.025	0.57 ± 0.031	1.05 ± 0.036	<0.0001
Feeling tense or keyed up	(Mean ± SEM)	1.39 ± 0.027	1.15 ± 0.038	1.57 ± 0.037	<0.0001
Spells of terror or panic	(Mean ± SEM)	0.46 ± 0.021	0.23 ± 0.022	0.63 ± 0.032	<0.0001
Feeling so restless you couldn’t sit still	(Mean ± SEM)	0.66 ± 0.022	0.41 ± 0.026	0.84 ± 0.033	<0.0001
**Suicide attempt/deliberate self-harm**					
SUIC	(%)	12.30%	4.80%	17.80%	<0.0001
**Hopelessness**					
BHS	(Mean ± SEM)	NA	0.198 ± 0.005	NA	
**Neuroticism, impulsiveness**					
BFI Neuroticism	(Mean ± SEM)	3.13 ± 0.019	2.82 ± 0.026	3.36 ± 0.025	<0.0001
IVE Impulsiveness	(Mean ± SEM)	0.34 ± 0.005	0.3 ± 0.007	0.37 ± 0.006	<0.0001
BIS Nonplanning Impulsivity	(Mean ± SEM)	NA	3.273 ± 0.013	NA	
BIS Motor Impulsivity	(Mean ± SEM)	NA	3.0 ± 1.786	NA	
BIS Attentional Impulsivity	(Mean ± SEM)	NA	3.625 ± 1.995	NA	
**Genotype**					0.158
TT	(N)	750	324	426	
TC	(N)	1084	453	631	
CC	(N)	449	198	251	
MAF	(%)	43.40%	44%	43.30%	

BFI, Big Five Inventory; BGR, background questionnaire; BHS, Beck Hopelessness Scale; BIS, Barratt Impulsiveness Scale; BSI, Brief Symptom Inventory (score range 1–4); BSI-ANX, BSI anxiety score; BSI-DEP, BSI depression score; IVE, Eysenck Impulsivity, Venturesomeness and Empathy Questionnaire; Minor Allele Frequency; SEM, standard error of mean; MAF, minor allele frequency; SUIC, self-reported suicide attempt/deliberate self-harm.

### Depression, Anxiety and suicide attempt

**Figure 1:**
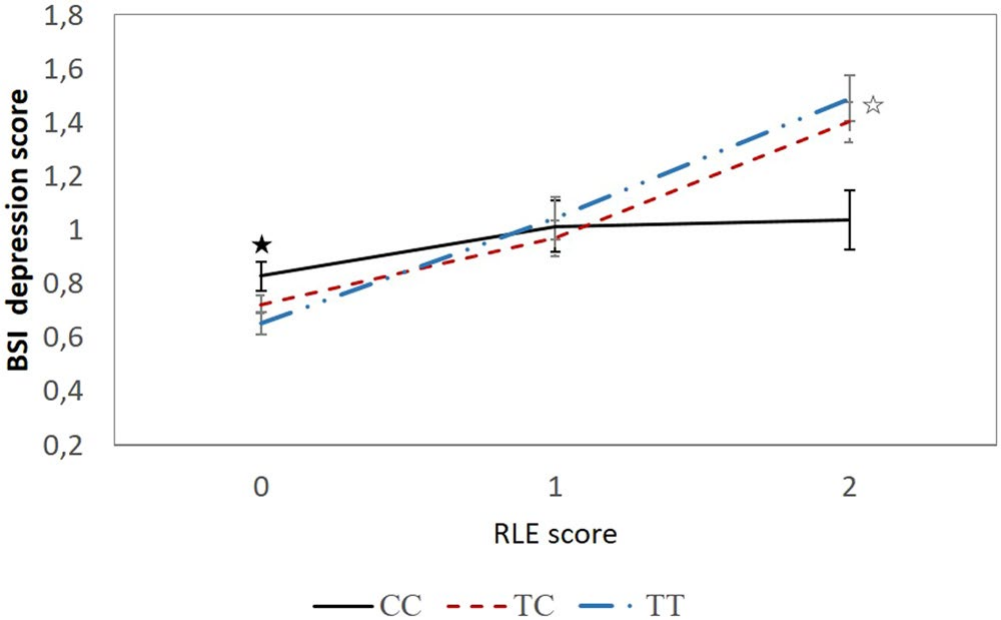
Significant interaction between recent negative life events (RLE) and *GABRA6* rs3219151 on current depression scores in the total population. Significant (p = 0.001) genetic interaction in mean BSI depression scores over RLE scores with standard error bars. Subjects carrying the T allele of GABRA6 rs3219151 showed higher increase in current depression scores when exposed to severe recent negative life events compared to those carrying the CC genotype. (Subject numbers in the RLE categories, respectively: CC genotype: RLE0: 288, RLE1: 92, RLE2: 69; TC genotype: RLE0: 728, RLE1: 196, RLE2: 154; TT genotype: RLE0: 506, RLE1: 131, RLE2: 110; ★ indicates p = 0.03 CC vs TT; ☆ indicates p = 0.08 CC vs TC, and p = 0.009 CC vs TT, pairwise comparisons for visualisation of results.) RLE0: 0–1 RLE; RLE1: 2 RLE; RLE2: 3 or more RLE (used only for display purposes). BSI: Brief Symptom Inventory; RLE: recent negative life events.

**Figure 2:**
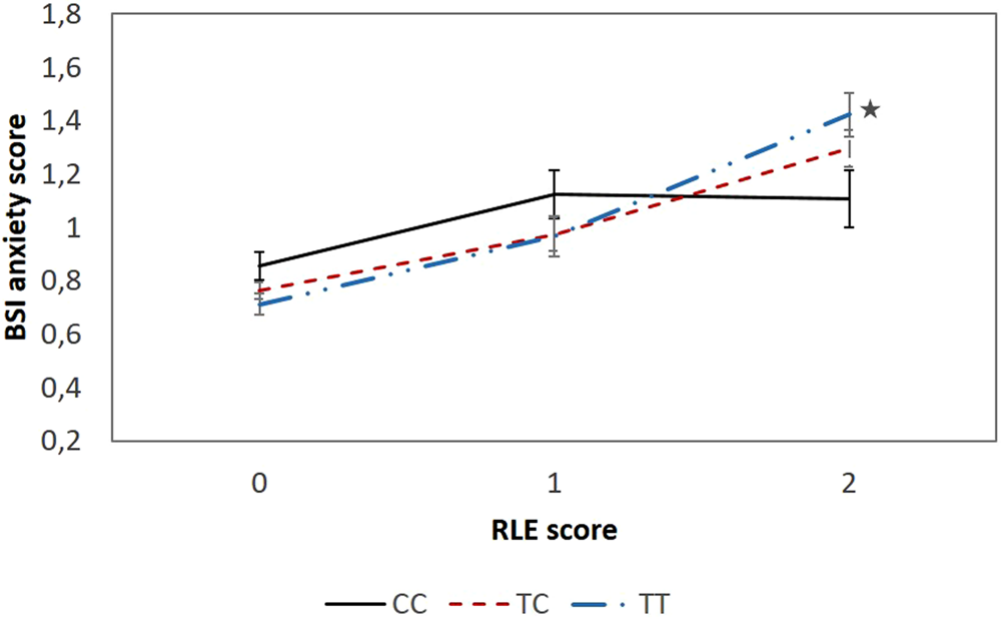
Significant interaction between recent negative life events (RLE) and *GABRA6* rs3219151 on current anxiety scores in the total population. Significant (p = 0.003) genetic interaction in mean BSI anxiety scores over RLE scores with standard error bars. Subjects carrying the T allele of GABRA6 rs3219151 showed higher increase in current anxiety scores when exposed to severe recent negative life events compared to those carrying the CC genotype (Subject numbers in the RLE categories, respectively: CC genotype: RLE0: 288, RLE1: 92, RLE2: 69; TC genotype: RLE0: 728, RLE1: 196, RLE2: 154; TT genotype: RLE0: 506, RLE1: 131, RLE2: 110; ★ indicates p = 0.016 CC vs TT; pairwise comparisons for visualisation of results.). RLE0: 0–1 RLE; RLE1: 2 RLE; RLE2: 3 or more RLE (used only for display purposes). BSI: Brief Symptom Inventory; RLE: recent negative life events.

The *GABRA6* SNP rs3219151 showed no significant main effect on DEP, SUIC or BSI-DEP and BSI-ANX in the combined Level 1 cohort (Table 2). However, rs3219151 showed significant association with BSI-DEP (p = 0.001, FDR-q = 0.008) and BSI-ANX (p = 0.003, FDR-q = 0.012) scores when interacting with RLE score (Table 2). The minor homozygote (CC) group has lower mean symptom scores than the other two genotype groups in individuals with RLE scores equal or over 3 for both BSI-DEP (Fig. 1) and BSI-ANX (Fig. 2). An average of 27% increase in BSI-DEP and BSI-ANX was found in individuals with CC genotype compared to an average 114% with TT genotype when high (equal or over 3) and mild (0–1) RLE groups were compared (Figs 1 and 2). This means that the effect of high RLE score on either BSI-DEP or BSI-ANX was in average 4 times higher in TT compared to CC genotype individuals.

**Table 2:** Main effects and interactions with recent negative life events (RLE) of *GABRA6* rs3219151 on BSI depression (BSI-DEP) and anxiety scores (BSI-ANX), lifetime depression (DEP) and self-reported suicide attempt/deliberate self-harm (SUIC) in the total population and sensitivity analysis.

Total population	BSI-DEP				BSI-ANX				DEP					SUIC				
*GABRA6* rs3219151	BETA	SE	STAT	P	BETA	SE	STAT	P	OR	L95	U95	STAT	P	OR	L95	U95	STAT	P
Main effect	0.028	0.027	1.051	0.293	0.040	0.026	1.505	0.133	1.029	0.905	1.169	0.434	0.664	0.901	0.750	1.083	−1.107	0.268
Interaction with RLE	−0.068	0.020	−3.397	0.001	−0.060	0.020	−2.998	0.003	0.890	0.803	0.988	−2.189	0.029	0.894	0.785	1.019	−1.682	0.093
Sensitivity analysis*																		
Interaction with RLE	−0.064	0.021	−3.131	0.002	−0.054	0.020	−2.678	0.007	0.899	0.804	1.005	−1.872	0.061	NA	NA	NA	NA	NA

We tested additive models. BSI, Brief Symptom Inventory; BSI-ANX, BSI anxiety; BSI-DEP, BSI depression; DEP, lifetime depression; RLE, recent negative life events; SUIC, self-reported suicide attempt/deliberate self-harm.

*Reanalysis of significant results after excluding subjects who reported bipolar disorder, schizophrenia or obsessive-compulsive disorder.

The rs3219151xRLE interaction effect was nominally significant on DEP (p = 0.029, FDR-q = 0.077) and showed a trend on SUIC (p = 0.093, FDR-q = 0.186) but none of them survived correction for multiple testing.

To help validate these results the two populations, Manchester and Budapest were analysed separately. The interaction effects on symptom scores were significant for both Manchester (BSI-DEP: p = 0.019; BSI-ANX: p = 0.043) and Budapest (BSI-DEP: p = 0.008; BSI-ANX: p = 0.013). Both populations showed similar patterns of change in mean BSI-DEP (Supplementary Fig. 1) and BSI-ANX (Supplementary Fig. 2) scores with increasing RLE score. Again, individuals with the minor CC genotype showed a slower increase in mean BSI-DEP and BSI-ANX scores with increasing stress exposure than the other genotype groups.

The rs3219151 x RLE interaction effect was nominally significant on DEP (p = 0.013) and on SUIC (p = 0.037) in the Manchester population but no significant interaction can be seen in Budapest (DEP: p = 0.699; SUIC: p = 0.679) probably because the Budapest cohort represented an average population with low prevalence of DEP (21.4%) and SUIC (4.8%), while the Manchester cohort was enriched with subjects suffering from DEP (56.2%) and reporting SUIC (17.8%).

### Sensitivity analysis

The genotype x RLE interactions remained statistically significant after exclusion of individuals who reported a history of manic or hypomanic episodes, psychotic symptoms, or obsessive-compulsive disorder from the analysis (p = 0.002 for BSI-DEP and p = 0.007 for BSI-ANX (Table 2). This suggests the interactions are not explained by major effects in less common disorders associated with abnormal mood.

### Post hoc analysis of BSI items

To determine factors that might increase the risk of suicide, the items of BSI-DEP and BSI-ANX were tested separately in the combined population (Table 3). Significant interaction effects were apparent for specific elements of suicide risk such as directly suicide-related thoughts (thoughts of ending your life: p = 0.004, thoughts of death or dying: p = 0.002), hopelessness related thoughts (feeling hopeless about the future: p = 0.019), restlessness and agitation (feeling so restless you could not sit still: p = 0.0008, feeling tense or keyed up: p = 0.012), insomnia (trouble falling asleep: p = 0.013), anhedonia (feeling no interest in things: p = 0.002), and acute anxiety attacks (spells of terror and panic: p = 0.00003). In addition, symptoms of depression secondarily related to suicide also showed significant association with rs3219151 x RLE interaction including depressed mood (feeling blue: p = 0.008) and depression-related thought contents (feelings of worthlessness: p = 0.004, feeling of guilt: p = 0.002).

**Table 3:** Post hoc analysis of association of *GABRA6* rs3219151 in interaction with recent negative life events on individual BSI items in the total population.

*GABRA6* rs3219151 in interaction with RLE				
*BSI-DEP*	BETA	SE	STAT	P
Thoughts of ending your life	−0.060	0.021	−2.895	0.004
Poor appetite	−0.007	0.022	−0.331	0.741
Feeling lonely	−0.059	0.031	−1.932	0.054
Feeling blue	−0.078	0.029	−2.646	0.008
Feeling no interest in things	−0.083	0.027	−3.052	0.002
Trouble falling asleep	−0.075	0.030	−2.482	0.013
Feeling hopeless about the future	−0.069	0.029	−2.344	0.019
Thoughts of death or dying	−0.084	0.027	−3.104	0.002
Feelings of worthlessness	−0.080	0.028	−2.883	0.004
Feelings of guilt	−0.084	0.027	−3.101	0.002
***BSI-ANX***				
Nervousness or shakiness inside	−0.048	0.029	−1.701	0.089
Suddenly scared for no reason	−0.033	0.024	−1.348	0.178
Feeling fearful	−0.034	0.027	−1.287	0.198
Feeling tense or keyed up	−0.072	0.029	−2.521	0.012
Spells of terror or panic	−0.095	0.023	−4.219	<0.0001
Feeling so restless you couldn’t sit still	−0.081	0.024	−3.364	<0.001

BSI, Brief Symptom Inventory; BSI-ANX, BSI anxiety; BSI-DEP, BSI depression; RLE, recent negative life events.

Two items showed independent nominally significant replication in the two study cohorts, namely thoughts of ending your life (Budapest p = 0.025; Manchester p = 0.047) and spells of terror and panic (Budapest p = 0.014; Manchester p = 0.001). Regarding all items and cohorts the T allele increased the risk to report the given symptom (Supplementary Table 1).

### Post hoc analysis of additional risk factors for suicide

**Figure 3:**
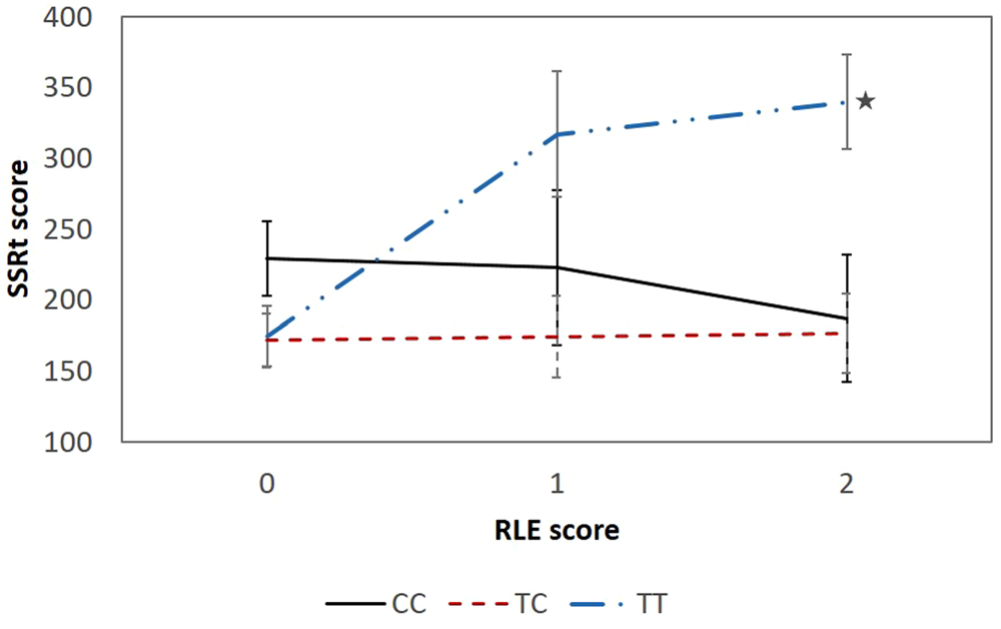
Significant interaction between recent negative life events (RLE) and *GABRA6* rs3219151 on current stop task reaction time (SSRT) scores in the Manchester Level 2 population. Significant (p = 0.0009) genetic interaction in mean SSRT over RLE scores with standard error bars. Subjects carrying the TT genotype of GABRA6 rs3219151 showed higher increase in SSRT when exposed to recent negative life events compared to those carrying the C allele (subject numbers in the RLE categories, respectively: CC genotype: RLE0: 26, RLE1: 6, RLE2: 9; TC genotype: RLE0: 51, RLE1: 21, RLE2: 23; TT genotype: RLE0: 39, RLE1: 9, RLE2: 16; ★ indicates p = 0.037 TC vs TT; pairwise comparisons for visualisation of results). RLE0: 0–1 RLE; RLE1: 2 RLE; RLE2: 3 or more RLE (used only for display purposes). BSI: Brief Symptom Inventory; RLE: recent negative life events.

We tested personality factors neuroticism (NEUR) and impulsiveness (IMP)  to determine whether the T allele has main effect or gene x environmental effect on them that might be related to increased suicide risk. Furthermore, we tested specific facets of impulsivity in the Budapest sample (motor - mIMP, cognitive - cIMP, and non-planning –nIMP) and in the Manchester Level 2 sample (STOP task SSRT (stop signal reaction time)). None of them showed any significant direct association with the T allele but in interaction with RLE a strong association was found on the STOP task SSRT (p = 0.0009, Table 4) with the TT genotype carriers showing increased stop signal reaction time if moderate or high RLE were present (Fig. 3). In addition, a weak, nominally significant association was found on NEUR (p = 0.031) in the combined population. Finally, hopelessness was specifically tested in the Budapest sample, because it can reliably predict suicide risk^18^. Rs3219151 showed no main effect but a significant rs3219151 x RLE interaction on hopelessness scores (p = 0.002, Table 4).

**Table 4:** Main effects and interactions with recent negative life events (RLE) of *GABRA6* rs3219151 on neuroticism, impulsiveness, hopelessness and STOP task reaction time in the relevant populations.

*GABRA6 rs3219151*	Total Population				Budapest				Manchester			
Main effect	BETA	SE	STAT	P	BETA	SE	STAT	P	BETA	SE	STAT	P
BFI Neuroticism score	0.039	0.026	1.529	0.127	0.052	0.038	1.382	0.168	0.024	0.035	0.695	0.487
IVE Impulsivity score	0.007	0.007	1.028	0.304	0.001	0.01	0.121	0.904	0.009	0.009	0.953	0.341
**Interaction with RLE**												
BFI Neuroticism score	−0.043	0.02	−2.156	0.031	−0.061	0.031	−1.944	0.052	−0.033	0.026	−1.294	0.196
IVE Impulsivity score	−0.009	0.005	−1.645	0.1	−0.018	0.008	−2.197	0.028	−0.003	0.007	−0.493	0.622
**Main effect**	**Budapest**				**Interaction with RLE**				**Budapest**			
BIS Nonplanning Impulsivity	0.008	0.019	0.419	0.676	BIS Nonplanning Impulsivity				−0.024	0.016	−1.565	0.118
BIS Motor Impulsivity	−0.004	0.014	−0.287	0.774	BIS Motor Impulsivity				−0.015	0.012	0.203	0.203
BIS Attentional Impulsivity	−0.018	0.017	−1.03	0.303	BIS Attentional Impulsivity				−0.01	0.014	−0.674	0.501
Hopelessness	−0.0003	0.008	−0.043	0.965	Hopelessness				−0.02	0.006	−3.028	0.002
**Main effect**	**Manchester Level 2**				**Interaction with RLE**				**Manchester Level 2**			
STOP task stop reaction time	−14.41	13.84	−1.041	0.299	STOP task stop reaction time				−30.42	9.033	−3.367	< 0.001

BFI, Big Five Inventory; BIS, Barratt Impulsiveness Scale; IVE, Eysenck’s Impulsivity, Venturesomeness and Empathy Questionnaire; RLE, recent negative life.

### In silico functional analysis

The GABRA6 SNP rs3219151 is located within the three prime untranslated region (3′UTR) of the *GABRA6* gene. *In silico* functional analysis predicted that rs3219151 was included in the target region for 4 miRNAs, hsa-miR-1178 (allele = T, score = 141, energy = −13.31; allele = C, score = 141, energy = −11.99) and hsa-miR-485-5p (allele = T, score = 147, energy = −15.83; allele = C, score = 142, energy = −14.03) where predicted to bind for either allele and hsa-miR-600 (allele = T, score = 155, energy = −14.36) and hsa-miR-920 (allele = C, score = 140, energy = −22.5) only one. Expression data was available for 3 of the miRNAs (hsa-miR-1178, hsa-miR-485 and hsa-miR-600) with all showing expression in the cerebellum and adrenal gland. Conservation score is PhyloP = 0.05.

**Figure 4:**
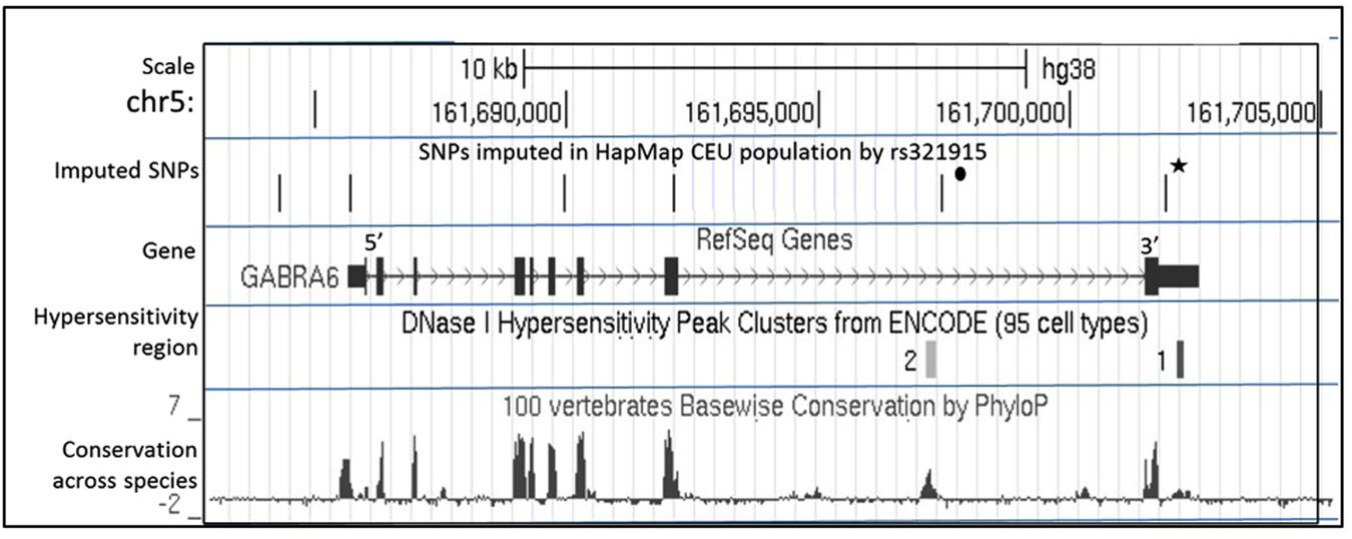
rs3219151 (*) located in the 3′UTR of GABRA6 imputes for 5 additional SNPs in the Caucasian HapMap CEU population. The imputed SNPs in order of appearance from left to right are rs1992646, rs3811995, rs3811992, rs13184586, rs13172914, with rs3219151 in the far right. Sequence conservation across species is shown as is the presence of DNase I hypersensitivity regions that are often associated with transcriptional regulation. Imputed SNP rs13172914 (•) is located just adjacent to a conserved region with potential transcriptional activity. Analyses were done using UCSC Genome browser on Human Dec. 2013 (GRCh38/hg38) with standard settings on all selected options.

**Figure 5:**
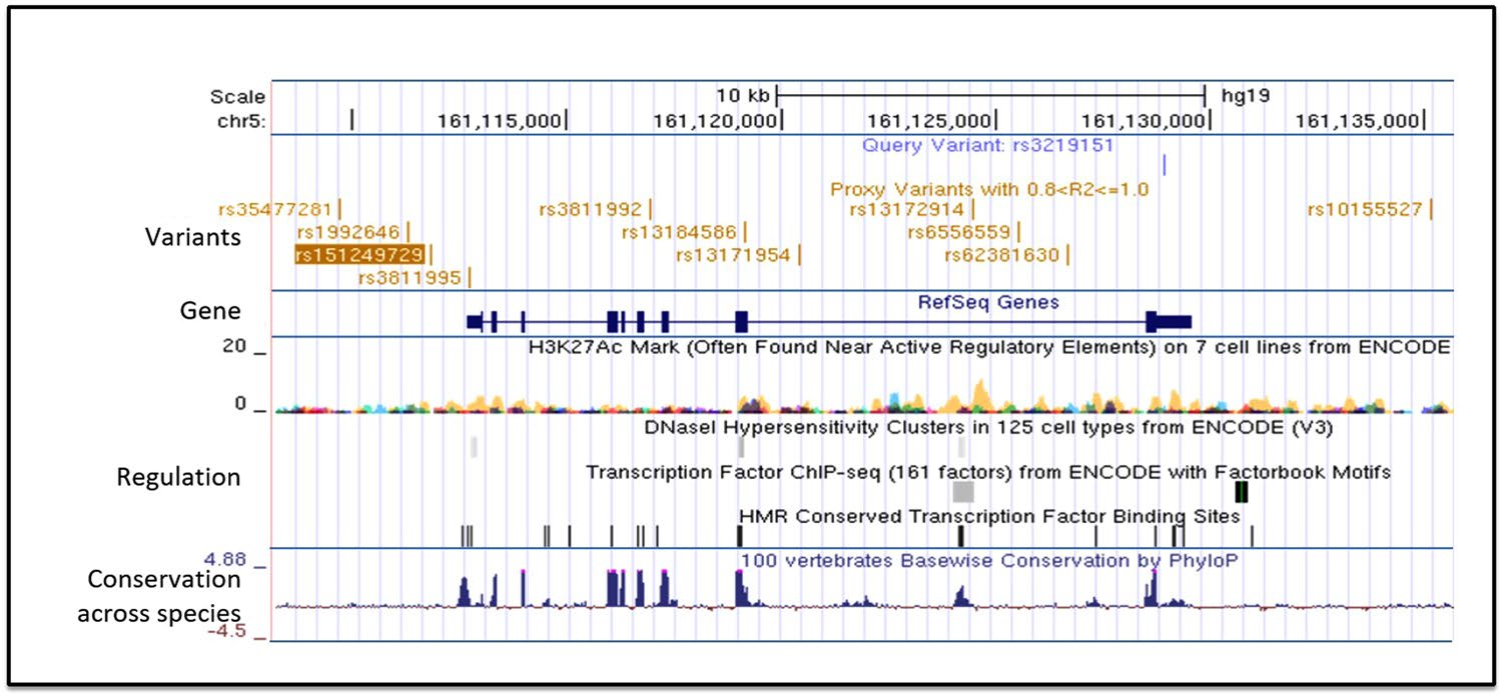
rs3219151, which is located in the 3′UTR of GABRA6 imputes for a total of 9 SNPs and 2 indels (rs151249729 and rs35477281) were in linkage disequilibrium (r2 > 0.8) with rs3219151 in the 1000 genome projects British in England and Scotland (GBR) sample. Sequence conservation across species is shown, as are DNase I hypersensitivity regions and H3K27Ac histone marks which are often associated with transcriptional regulation. Analysis is shown using UCSC Genome Browser on Human Feb. 2009 (GRCh37/hg19).

Rs3219151 is located between 2 regions of high conservation and can be used to impute the genotype for 5 additional SNPs (rs1992646, rs3811995, rs3811992, rs13184586, rs13172914) with a high level of accuracy (r2 > 0.8) in the Caucasian HapMap CEU population. The imputed SNP rs13172914 is located adjacent to a conserved DNase I hypersensitivity region suggesting that it is in or close to a region of potential transcriptional activity (Fig. 4). In addition, a total of 9 SNPs (rs1992646, rs3811995, rs3811992, rs13184586, rs13171954, rs10155527, rs13172914, rs6556559, rs62381630 and rs35477281) and 2 indels (rs151249729 and rs35477281) were in linkage disequilibrium (r2 > 0.8) with rs3219151 in a British population sample. These SNPs span the length of the GABRA6 gene and include rs3811995 located in the 5′UTR of GABRA6 and rs13184586 a synonymous SNP located in exon 8 (Fig. 5). One SNP rs13171954 located in the final intron of GABRA6 was predicted to affect the binding of several transcription factors of the foxhead box family by RegulomeDB, with the minor G allele disrupting the site. All analyses were done using UCSC Genome Browser on Human Dec. 2013 (GRCh38/hg38) with standard settings on all selected options.

## Discussion

We identified a complex interaction between *GABRA6* rs3219151 T allele and recent life stress in multiple phenotypes associated with suicidal behaviour converging to a significant suicide risk. As no main effects of rs3219151 on any investigated phenotypes were revealed this variant allele likely plays a role in mediating the effects of recent stress in the emergence of suicidal behaviour. After exposure to recent negative events, however, presence of the *GABRA6* rs3219151 T allele increased risk of current depression (BSI-DEP) and anxiety (BSI-ANX), as well as specific elements of suicidal risk including directly suicide-related thoughts, hopelessness, restlessness and agitation, insomnia, and acute anxiety. We also found a strong association between cognitive impulsivity as measured by the STOP task and the T allele in interaction with stress exposure, while neuroticism and trait impulsiveness showed no association. Our data thus suggest a possible role of the T allele in stress-related suicide risk as a result of the constellation of several independent suicide-related phenotypes.

There is increasing evidence that response to environmental stressors has strong genetic determinants. In our study we observed no significant direct effect of the *GABRA6* T allele on any of our investigated variables, suggesting that this variant plays a role in stress-induced psychopathology. Similarly, we found no signs of gene-environment correlation, as the investigated genotype showed no associations with any of the demographic or lifestyle measures investigated in our study. A strong increase in depression and anxiety scores were, however, observable with increasing recent life stress in carriers of the T allele.

The GABA system interacts with stressors playing a role in brain-level stress control^9^ inhibiting the HPA axis via GABA-A receptors in CRH neurons in the hypothalamus including the PVN^19^ and attenuating stress response^10^). Several studies have found associations between GABA-A subunit polymorphisms and stress reactivity. The GABA-A receptor alpha subunit family contains several isoforms including the alpha6 isoform encoded by a gene in chromosome 5q34.

Rs3219151 is located within the 3′ untranslated region (3′UTR) of *GABRA6* which contains regulatory regions that post-transcriptionally influence gene expression, and has previously been predicted to alter at least one micro-RNA (miRNA) binding site^20^. In silico analysis supported this with hsa-miR-600 and hsa-miR-920 predicted to bind with the T and C alleles respectively, supporting the idea that rs3219151 alters miRNA regulation. miRNAs play a role in regulating gene expression levels including playing a key role in brain development, epigenetic programming and stress response^21^. Exposure to chronic stress has been shown to have long lasting effects on miRNA expression levels, which is of interest because alterations in the expression patterns of miRNAs have been reported in a number of psychiatric conditions^22^. The role of miRNA in regulating *GABRA6* expression has also already been shown with a previous study showing miR-138-2 reduces *GABRA6* expression by 30%. This study also showed an association between non-acrophobic panic disorder and the miR-138-2 tagging SNP rs12921781 in a Spanish cohort^23^.

Additionally, rs3219151 is in linkage disequilibrium with 9 additional SNPs and 2 indels in a British population (Fig. 5) and imputes for 5 additional SNPs in a Caucasian HapMap CEU population some of which have potential functional effects (Fig. 4)^14^. One intronic SNP rs13171954 is predicted to affect the binding of several foxhead box family transcription factors, with the minor G allele disrupting the site, and a second SNP rs3811995 is located in the 5′ untranslated region, while the imputed rs13172914 is in or close to a region of potential transcriptional activity (Fig. 4).

Previous results suggested an association between hormonal and psychological stress response and the *GABRA6* gene. Variations in *GABRA6* and specifically rs3219151 T allele was found to be related to higher baseline salivary cortisol levels^13^ and higher ACTH, cortisol and blood pressure increase upon stress exposure in several studies^14,24,25^. These results suggest that decreased inhibition of the HPA axis due to GABA deficits may contribute to increased hormonal and physiological stress response^14^ which is well-known to be associated with risks of developing mental health problems including depression and suicide. Our present results indicating increased maladaptive psychological responses including depression and anxiety symptoms following stress exposure in *GABRA6* T carriers support and extend findings that this genetic variant may mediate the effects of stress.

In our study those carrying the T allele showed a significantly larger increase in the risk of current depression and anxiety after exposure to recent negative life events compared to CC carriers, while no such increase was observable in the absence of recent stress. Investigating our two subsamples separately, lifetime depression also showed a nominally significant association with the T allele in interaction with recent stress in the Manchester but not in the Budapest subsample likely due to the lower prevalence of depressed subjects in this latter subsample.

Previous research points to the possible involvement of GABA in the propensity of anxious and fearful traits and temperaments via its GABA-A receptor-mediated inhibitory effects with even minor genetic variation possibly contributing to an alteration in anxiety-related traits and individual differences in threat-related responses^26,27^. Specifically, *GABRA6* TT carriers were shown to exhibit increased harm avoidance and anticipatory worry^26,28^, and T carrier panic disorder patients showed increased reaction to fearful faces in an fMRI study^24^ thus suggesting an association between *GABRA6* and different aspects of anxiety^14,24,25^.

Similarly, there is increasing support for state-dependent GABA deficits in major depression^29–31^ including lower CSF, plasma and cortical GABA levels in *in vivo* MR studies^32–34^. Post-mortem results, however, although suggesting GABA dysfunction^35^, are more equivocal with prefrontal GABA levels showing an inverse association with depression severity in some studies^36^ and no GABA-related alterations in others^16^. Several antidepressants, anticonvulsants with antidepressant effects and electroconvulsive therapy increase GABA function^37–39^, and chronic antidepressant treatment has been demonstrated to normalize GABA (and glutamate) levels suggesting the possible involvement of GABA in antidepressant action^40^. The observed alteration of GABA function in major depression may be related to altered GABA-A receptor function. Specifically, the 5q33-35 area that encodes several GABA-A receptor subunits including *GABRA6*, *GABRA1* and *GABRG2* subunit genes and its corresponding area in mice was found to be related to depression-like behaviour^35^. Interestingly, the AA genotype of rs1992647, which is located downstream of *GABRA6* and tags for other SNPs within the gene, in an interaction with the environment showed association with nonresponse to antidepressant treatment^41^. Our results concerning the association of the *GABRA6* T allele in interaction with the environment on current and, to a limited extent, lifetime depression are in line with and in support of these previous findings.

While no main effect of the T allele on self-reported suicide attempts was identified, and we only found a trend in interaction with recent negative life events in the combined sample, there was a nominally significant rs3219151xRLE interaction effect in the Manchester but not the Budapest subsample probably related to the much higher prevalence of suicide in this former subsample. However, we detected strong associations with multiple possible predictors, markers and phenotypes related to suicide risk including suicidal and death-related thoughts, hopelessness^42^, restlessness and agitation^43^, insomnia^44^ as well as feelings of panic and acute anxiety^45^. While neuroticism showed only a weak nominally significant association in interaction with RLE, and trait-impulsiveness showed no association with the T allele either directly or in association with recent stress, a strong association was found in interaction with RLE between the T allele and cognitive impulsiveness measured by the STOP task.

A number of previous studies have investigated the role of GABA transmission and GABA genes in suicide but are inconclusive and contradictory^4^. Differential expression of GABA-A receptor subunits in prefrontal and limbic regions were reported in suicide and MDD^15,46,47^. Lower GABA alpha1, alpha3, alpha4 and delta subunit mRNA expression has been reported in the frontopolar cortex^16^, and modest but significant differences in alpha4 and delta subunit expression were found in the hippocampus and amygdala^17^ in depressive suicide victims. Furthermore, dysregulation in GABA-A receptor subunit mRNA integration and coordination, which may affect their configuration into a functional receptor, has been demonstrated in the frontopolar and dorsomedial prefrontal cortex, hippocampus and amygdala in suicide victims^16,17^ suggesting a dysregulation between GABA-A subunit genes and disturbed transcriptional-level coordination of the regulation of subunit expression^16^. Altered alpha subunit ratios may influence duration of inhibitory currents affecting inhibitory tone and change network timing patterns leading to a dysbalance between tonic and phasic inhibition in the frontal cortex, this could have behavioural and psychopathological consequences^16,17^. In another study significant GABA-A expression differences between depressed and non-depressed suicide victims were found in all limbic regions pointing to GABA dysfunction in the limbic system in depression and suicide, especially *GABRA1* and *GABRB1* ^48^. In a study investigating gene expression in 17 brain areas in depressed and nondepressed suicide victims and controls, they found that suicidal depressives exhibited an upregulation of a large number of GABA receptor subunit genes^15^. Our results indicating an association between the *GABRA6* gene and past suicidal behaviour as well as several suicide risk-related phenotypes are in line with the above results suggesting the involvement of GABA-A receptor subunit variations in suicide risk.

It should be mentioned that in our study specific elements of suicide risk show a great overlap with symptoms of depression. It is well-known that depression is one of the major contributors to suicide risk and there is also a partial overlap between the genetics of major depression and suicide^49^. Several studies investigating suicidal behaviour and also those studies focusing on the association between GABA system and suicide point out that further studies are needed to differentiate those suicidal risks associated with the investigated genetic variant which are specific for suicide but not major depression^17^. However, the robust association in our present study between rs3219151 x RLE and BSI-depression items “thoughts of death or dying”, “feeling no interest”, “feeling of guilt”, especially coupled with BSI-anxiety items related to agitation, restlessness, and anxiety attacks, as well as with hopelessness yields a constellation that argues for the observed association being specific for suicide risk.

In order to investigate whether the *GABRA6* T allele directly or in GxE interactions is associated with traits and cognitive phenotypes influencing suicide risk we tested neuroticism, trait impulsiveness and the STOP task reaction time in subsamples. Interestingly, we found only a nominal association for neuroticism in spite of earlier studies where *GABRA6* was found to be related to neuroticism and harm avoidance^14,24,25^.

Similarly, no association between rs3219151 and trait impulsiveness which is an important contributor to certain forms of suicidal behaviour was reported in our study. However, a highly significant association was reported in interaction with RLE for cognitive impulsiveness as measured by increased reaction times in T carriers in the STOP task reflecting increased latency in the ability to inhibit an already initiated response. Through its crucial role in prefrontal-limbic cortex circuitry which plays a role in affective processing and behavioural inhibition, GABA appears to have an inhibitory effect on affectively-based impulsive behaviours^50^. However, cerebrospinal fluid and peripheral GABA concentration showed a controversial association with impulsiveness, either a direct or a reverse correlation have been described^50,51^. Similarly, while several GABA-enhancing treatments such as lithium, valproate or carbamazepine have been shown to decrease impulsiveness and impulsiveness-related behaviour, benzodiazepines increased impulsiveness in several studies^50^. Previously association of *GABRA6* variants have not been investigated in association with impulsiveness, so our results concerning an association between rs3219151 T allele and increased neurocognitive impulsivity (being slower to inhibit already initiated responses) in the context of greater life stress is a novel finding possibly paving the way for further studies delineating the controversial role of GABA in impulsiveness.

Several limitations of our study need to be mentioned. We applied a cross-sectional approach and all phenotypic measures were assessed based on self-report without psychiatric screening, although assessment of lifetime and current depression and anxiety was later validated in a subsample using SCID, MADRS and the Clinical Anxiety Scale^52^. Assessment of recent negative life events was also based on self-report. Furthermore, suicidal behaviour is hard to investigate due to its relatively low prevalence in the general population, and those already having committed suicide were naturally missing from our retrospectively evaluated sample. Several of our investigated trait and state-like phenotypes including impulsiveness and current depression may also influence willingness to participate in such studies and thus bias the sample.

Given previous data implicating the T allele of the *GABRA6* gene in association with heightened stress response, we interpreted our findings from the aspect of the T allele being a risk allele. However, our data can also be interpreted as the CC genotype, which has the lowest frequency, being protective against the increasing depression and anxiety observed with increasing life events exposure. Nevertheless, this does not influence our finding of the association of the GABRA6 rs3219151 with elements of suicide risk phenotype.

In summary we report in our study that *GABRA6* T allele plays an important role in mediating the effects of recent stress in the development of suicidal risk-related and possibly suicide-predictor phenotypes. These data provide evidence that stress-induced suicide risk may be elevated in T allele carriers, indicated by a constellation of elements associated with suicide risk including suicidal and death related thoughts, hopelessness and cognitive impulsiveness.

## Methods

The study was approved by the local Ethics Committees (Scientific and Research Ethics Committee of the Medical Research Council, Budapest, Hungary; and North Manchester Local Research Ethics Committee, Manchester, UK) and was carried out in accordance with the Declaration of Helsinki and all relevant rules and regulations as part of the NewMood study (New Molecules in Mood Disorders, Sixth Framework Program of the EU, LSHM-CT-2004-503474). All participants provided written informed consent.

### Study Cohorts

Subjects aged 18–60 years were recruited, as a population sample through general practices and a website, in two distinct geographic regions, Greater Manchester, United Kingdom and Budapest, Hungary. Full details of the recruitment strategy and criteria have been published previously^52–54^. For this study the experimental cohort was limited to unrelated individuals of self-reported Caucasian ancestry as this was the largest ethnic group, with successful genotyping producing a working European cohort of n = 2283 (Level 1 phase, for description see Table 1).

In addition, in Manchester a subset of the cohort and new participants underwent additional assessments to validate and extend self-report measures. From this, n = 204 participants were successfully genotyped with useful phenotypic data (see below) and included in the present study (Level 2, for description see Table 5).

**Table 5:** Description of the Level 2 population.

Population size	(N)	204
**Demographics**		
Gender	(% Male)	31.40%
Age	(Mean ± SEM) (range)	33.68 ± 0.784 (18–60)
**Recent negative life events**		
RLE-L2	(Mean ± SEM)	1.53 ± 0.110
**Prepotent behavioural response inhibition**		
SSRT (ms)	(Mean ± SEM)	201.37 ± 9.867
**Current Depression Score**		
MADRS	(Mean ± SEM)	5.56 ± 0.562
**Lifetime depression (based on SCID)**	(%)	59.2%
Remitted depression	(%)	40.8%
Partially remitted depression	(%)	5%
Current Depression	(%)	13.5%
**Genotype**		
TT	(N)	65
TC	(N)	98
CC	(N)	41
MAF	(%)	44.10%

Level 2 population: in Manchester a subset of the cohort and new participants underwent additional assessments to validate and extend self-report measures.

MADRS, Montgomery-Asberg Depression Rating Scale; MAF, Minor Allele Frequency; RLE-L2, Recent Negative Life Events in Level 2 population; SCID, Structured Clinical Interview for DSM-IV; SEM, Standard Error of Mean; SSRT, Stop signal reaction times in milliseconds.

### Investigated phenotypes

#### Phenotypic measures for Level 1

At Level 1 the participants filled out the study questionnaire pack and provided genetic samples. The questionnaire contained a background questionnaire (BGR)^53,54^, which included demographic, health and lifestyle measures, and an inventory of the individuals personal psychiatric history. For the primary analysis lifetime depression (DEP) was derived from this questionnaire, and was validated in the Level 2 phase based on the Structured Clinical Interview for DSM-IV (SCID-I/NP)^52,55^. In addition, self-reported suicide attempt and deliberate self-harm (SUIC) was analysed from the background questionnaire. Reported manic or hypomanic episodes, psychotic symptoms, or obsessive-compulsive disorder were used for sensitivity analysis (see below).

Psychiatric symptoms were measured using the Brief Symptom Inventory (BSI)^56^ by the depression subscale plus additional items to calculate depressive symptom scores (BSI-DEP), and by the anxiety subscale to derive anxiety symptoms (BSI-ANX)^57,58^. For depression and anxiety continuous weighted dimension scores were calculated (sum of items scored divided by the number of items completed). At a post hoc analysis we used the individual items of BSI (10 items for depression and 6 items for anxiety, scores ranging from 0 to 4) to demonstrate specific genetic effects.

To assess neuroticism (NEUR) we used the Big Five Inventory (BFI)^59^ neuroticism subscale. Impulsivity was measured by the Eysenck’s Impulsivity, Venturesomeness and Empathy Questionnaire^60^ impulsivity subscale (IMP), and in the Budapest sample (n = 975) we also used the Barratt Impulsiveness Scale (BIS-11) to specifically asses Motor Impulsiveness (mIMP), Cognitive Impulsiveness (cIMP), and Nonplanning Impulsiveness (nIMP)^61,62^. To specifically test suicide related phenotypes in the Budapest sample we measured Hopelessness by the Beck’s Hopelessness Scale (BHS)^63^. For these parameters continuous weighted dimension scores were calculated.

Recent negative life events (RLE), experienced in the last year, were measured by The List of Threatening Experiences (LTE)^64^ and a summary score was used in the analysis.

#### Post-hoc sensitivity analyses in Level 1

Depression and anxiety can occur concurrently with other mental health conditions. As previous studies have reports that *GABRA6* may also play a role in other mood disorders and schizophrenia^20,35^ individuals who reported manic or hypomanic episodes, psychotic symptoms, or obsessive-compulsive disorder (based on the Background Questionnaire) were not initially excluded from the main study. A sensitivity analysis was then carried out *post hoc* without these individuals to help determine the extent to which these individuals influenced any findings. This resulted in the exclusion of 41 and 116 individuals (4.2% and 8.9% of total population cohort) for the Budapest and Manchester cohorts respectively.

#### Phenotypic measures for Level 2

Based on the SCID-I/NP^35,55^ interview participants with any other psychiatric condition than major depressive disorder or anxiety disorders were excluded from the analyses. Current depressive symptoms were assessed by a trained investigator using the Montgomery-Åsberg Depression Rating Scale^65^. The depression severity ratings produced from these interviews were highly correlated (p < 0.001) with those from the self-report questionnaire^52^.

Prepotent behavioural response inhibition as a neurocognitive measure of impulsivity was assessed by the Stop task^66^. Stop signal reaction times (SSRT) were calculated as an outcome measure^67^ and analysed.

At this level an extended Life Events Questionnaire (LEQ) was used by adapting validated questionnaires^64,68,69^ and a sum score on recent (within the last year) negative life events (RLE-L2) was applied in the analysis. The original LTE showed significant and strong correlation with LEQ recent negative life events^52^.

### Genotyping

Genomic DNA was extracted using the Freeman *et al*.^70^ protocol from buccal mucosa cells collected by cytology brush (Cytobrush plus C0012; Durbin PLC). Genotyping was carried out using the IplexTM assay from Sequenoms MassARRAY technology (Sequenom, San Diego) following the manufacturer’s protocol (http://www.sequenom.com). A 15% replication of genotyping was built into the study design, from which an overall type I error rate of 0.016% was calculated.

### Statistical Analysis

Genetic statistical analysis was carried out using PLINK v1.07 (http://pngu.mgh.harvard.edu/purcell/plink/), including calculation of Hardy–Weinberg equilibrium and running regression models (logistic and linear, respectively) using additive genetic model for *GABRA6* rs3219151. Age at time of assessment, gender, and population (Budapest or Manchester) were used as covariates in all primary analyses and main effects of variables of the interaction terms were also included in all regression models. Significant skewing of the distribution of factors known to contribute to mental issues between genotypic groups was checked for gender, age, and RLE. In the primary analysis self-reported lifetime depression (DEP), self-reported suicide attempt/deliberate self-harm (SUIC), current depression (BSI-DEP) and anxiety (BSI-ANX) scores were used to examine the interaction between rs3219151 and stress. Recent negative life events (RLE) score was used as a proxy for stress. FDR Q values were calculated to correct for multiple testing during the primary analysis (http://qvalue.princeton.edu/)^71^ with q < 0.05 as significant.

As a post hoc test, to identify replications, the Budapest and Manchester subsamples were separately tested with the same method as above except that population was not covaried in the models. In-house written R-scripts^72^ were applied in the PLINK analysis of the Level 1 to perform the separate analyses in the Budapest and Manchester subsamples. Further variables were explored to identify factors that might increase suicide risk; neuroticism (NEUR) and impulsivity (IMP) were investigated in the Level1 combined population, Motor Impulsiveness (mIMP), Cognitive Impulsiveness (cIMP), Nonplanning Impulsiveness (nIMP), and Hopelessness (BHS) in the Budapest Level1 population, and behavioural inhibition (SSRT) in the Manchester Level2 population. For the post hoc tests nominal two-tailed p ≤ 0.05 was the significance threshold.

Descriptive statistics were calculated with IBM SPSS Statistics 23 (http://www.ibm.com/analytics/us/en/technology/spss/). Based on Quanto (http://biostats.usc.edu/Quanto.html) assuming an explained variance (R^2^) of 1% or odds ratio (OR) of 1.2 we have 99.77% and 99.10% power to detect additive genetic main effects respectively (p ≤ 0.05), or 99.80% and 99.99% power to capture gene x stress interactions respectively (p ≤ 0.05), in our combined cohort (n = 2283) for rs3219151. We also have 70.29% and 71.17% power to capture genetic main effects or gene x stress interaction respectively, that explains 3% variance (R^2^) in our Level 2 population (n = 204).

### *In silico* functional analysis

SNPs in linkage disequilibrium (LD) with rs3219151 (r2 > 0.8) were identified using the National Cancer Institute, Division of Cancer Epidemiology & Genetics’ LDLink tools (https://analysistools.nci.nih.gov/LDlink/?tab=ldproxy) with phase 3 data from the 1000 genome projects British in England and Scotland (GBR) sample. Additional assessment of potential functional impact of rs3219151 and any SNPs in LD with rs3219151 (r2 > 0.8) was carried out using SNP Function Prediction (FuncPred; National Institute of Environmental Health Sciences, North Carolina, USA; https://snpinfo.niehs.nih.gov/snpinfo/snpfunc.html) and mutationtaster (Charité, Berlin; http://www.mutationtaster.org/ChrPos.html). Cutoffs for miRanda miRNA prediction within FuncPred were score ≥ 140 and Gibbs free energy ≤ −7.0. Potential impact of the SNPs on known and predicted regulatory elements in the intergenic regions was investigated using the RegulomeDB database (Center for Genomics and Personalized Medicine, Stanford University; http://www.regulomedb.org/index) and ENCODE (Encyclopedia of DNA Elements) project data via the UCSC Genome Browser (University of California, Santa Cruz; http://genome.ucsc.edu/ENCODE/), with conservation at the SNP positions across 100 vertebrate species reported using basewise conservation (phyloP) score. Additional information on predicted miRNA regulation was obtained from miRBase (University of Manchester, UK; http://www.mirbase.org/search.shtml). All analyses were done using UCSC Genome Browser on Human version GRCh38/hg38 released Dec. 2013 with standard settings on all selected options.

### Data availability

The datasets generated during and/or analysed during the current study are available in the Figshare repository, https://figshare.com/s/fbd4e19a942aae3b2c09 and https://figshare.com/s/14462b7a1876a8279ca3.

## Electronic supplementary material

Supplementary Information
