# Supplementary material for: A new stress sensor and risk factor for suicide: the T allele of the functional genetic variant in the GABRA6 gene
Source: Sci Rep. 2017 Oct 10;7:12887. doi: 10.1038/s41598-017-12776-8 (PMC5635130; doi:10.1038/s41598-017-12776-8)
Supplement: Supplementary file 1 — Supplementary Information [file 41598_2017_12776_MOESM1_ESM.pdf]

Supplementary Information

A new stress sensor and risk factor for suicide: the T allele of the functional genetic variant in the GABRA6 gene

Xenia Gonda, Jane Sarginson, Nora Eszlari Peter Petschner, Zoltan G. Toth, Daniel Baksa, Gabor Hullam, Ian M Anderson, JF William Deakin, Gabriella Juhasz, Gyorgy Bagdy

Supplementary Table 1 Post hoc analysis of the interactions with recent negative life events (RLE) of *GABRA6* rs3219151 on individual BSI items in the two subpopulations

GABRA6 rs3219151 in interaction with RLE	Budapest				Manchester			
BSI-DEP	BETA	SE	STAT	P	BETA	SE	STAT	P
Thoughts of ending your life	***-0.066***	***0.029***	***-2.242***	***0.025***	***-0.056***	***0.028***	***-1.984***	***0.047***
Poor appetite	-0.026	0.033	-0.763	0.446	0.002	0.030	0.077	0.939
Feeling lonely	-0.042	0.048	-0.876	0.382	-0.071	0.040	-1.777	0.075
Feeling blue	-0.089	0.044	-2.037	0.042	-0.069	0.039	-1.758	0.079
Feeling no interest in things	-0.118	0.034	-3.411	0.001	-0.061	0.039	-1.569	0.117
Trouble falling asleep	-0.118	0.042	-2.794	0.005	-0.048	0.042	-1.159	0.247
Feeling hopeless about the future	-0.082	0.042	-1.947	0.052	-0.054	0.040	-1.353	0.176
Thoughts of death or dying	-0.055	0.040	-1.378	0.169	-0.104	0.037	-2.844	0.005
Feelings of worthlessness	-0.046	0.033	-1.382	0.167	-0.097	0.040	-2.410	0.016
Feelings of guilt	-0.053	0.037	-1.454	0.146	-0.101	0.038	-2.652	0.008
BSI-ANX								
Nervousness or shakiness inside	-0.079	0.047	-1.667	0.096	-0.034	0.036	-0.941	0.347
Suddenly scared for no reason	-0.037	0.035	-1.067	0.286	-0.030	0.033	-0.899	0.369
Feeling fearful	-0.065	0.037	-1.766	0.078	-0.016	0.037	-0.445	0.656
Feeling tense or keyed up	-0.113	0.045	-2.527	0.012	-0.054	0.038	-1.432	0.153
Spells of terror or panic	***-0.065***	***0.027***	***-2.455***	***0.014***	***-0.109***	***0.033***	***-3.328***	***0.001***
Feeling so restless you couldn't sit still	-0.040	0.032	-1.271	0.204	-0.104	0.034	-3.062	0.002

BSI, Brief Symptom Inventory; BSI-ANX, BSI anxiety; BSI-DEP, BSI depression; RLE, recent negative life events

Bold italics indicate significant results replicable in both subsamples.

Supplementary Figure 1 Significant interaction between recent negative life events (RLE) and *GABRA6* rs3219151 on current depression scores in the two subpopulations

a.

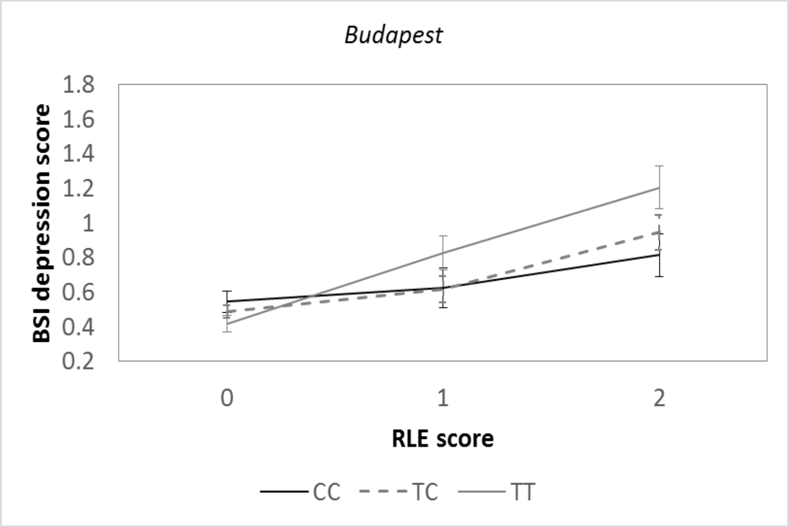

subject numbers in the RLE categories, respectively:

CC genotype: RLE0: 130; RLE1: 36; RLE2: 32

TC genotype: RLE0: 319; RLE1: 84; RLE2: 44

TT genotype: RLE0: 238; RLE1: 50; RLE2: 33

b.

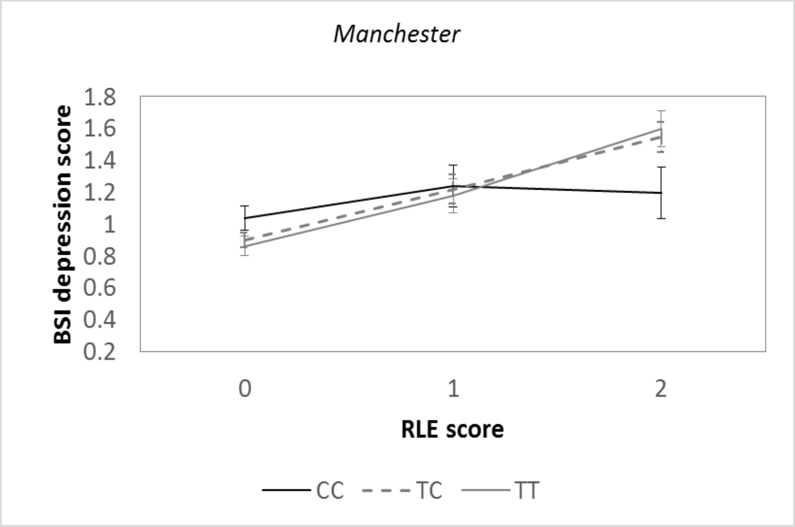

subject numbers in the RLE categories, respectively:

CC genotype: RLE0: 158; RLE1: 56; RLE2: 37

TC genotype: RLE0: 409; RLE1: 112; RLE2: 110

TT genotype: RLE0: 268; RLE1: 81; RLE2: 77

Significant (Budapest: p=0.008; Manchester p=0.019) genetic interaction in mean BSI depression score over RLE scores with standard error bars. Subjects carrying the T allele of GABRA6 rs3219151 showed higher increase in BSI depression score when exposed to severe recent negative life events compared to those carrying the CC genotype.

RLE0: 0-1 RLE; RLE1: 2 RLE; RLE2: 3 or more RLE (used only for display purposes).

BSI: Brief Symptom Inventory; RLE: recent negative life events.

Supplementary Figure 2 Significant interaction between recent negative life events (RLE) and *GABRA6* rs3219151 on current anxiety scores in the total two subpopulations

a. b.

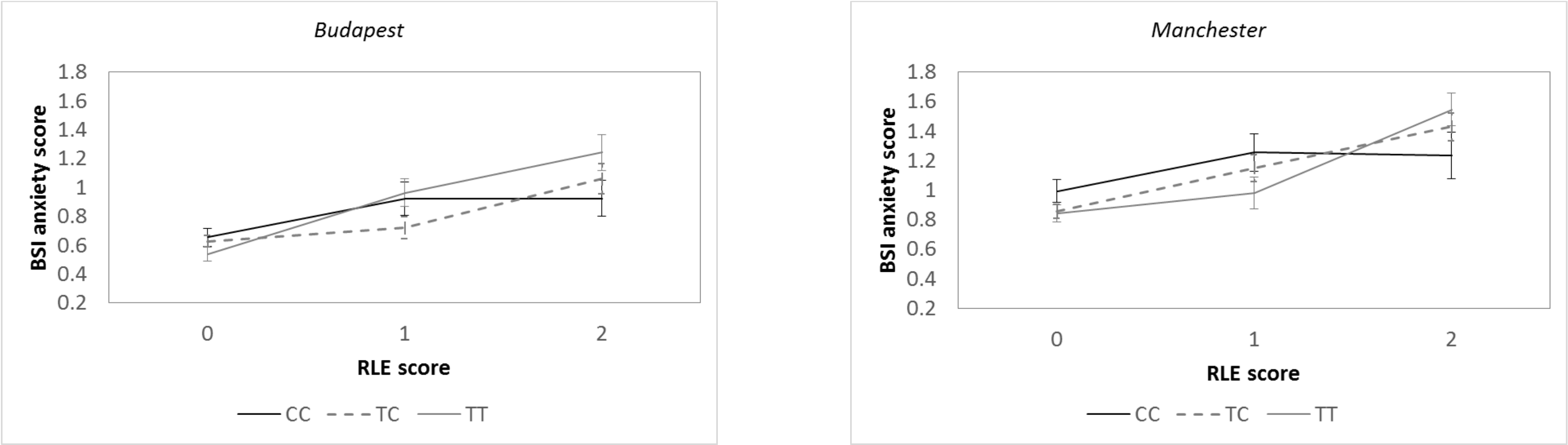

a. subject numbers in the RLE categories, respectively:
CC genotype: RLE0: 130; RLE1: 36; RLE2: 32
TC genotype: RLE0: 319; RLE1: 84; RLE2: 44
TT genotype: RLE0: 238; RLE1: 50; RLE2: 33

b. subject numbers in the RLE categories, respectively:
CC genotype: RLE0: 158; RLE1: 56; RLE2: 37
TC genotype: RLE0: 409; RLE1: 112; RLE2: 110
TT genotype: RLE0: 268; RLE1: 81; RLE2: 77

Significant (Budapest: p=0.013; Manchester p=0.043) genetic interaction in mean BSI anxiety score over RLE scores with standard error bars. Subjects carrying the T allele of GABRA6 rs3219151 showed higher increase in BSI anxiety score when exposed to severe recent negative life events compared to those carrying the CC genotype.

RLE0: 0-1 RLE; RLE1: 2 RLE; RLE2: 3 or more RLE (used only for display purposes).

BSI: Brief Symptom Inventory; RLE: recent negative life events.
